# *Lacticaseibacillus rhamnosus* GG lysate inhibits *Staphylococcus aureus* biofilm formation in human skin explants

**DOI:** 10.3389/fcimb.2026.1885754

**Published:** 2026-07-21

**Authors:** Abigail E. Elias, Cecile El-Chami, James Bagnall, Andrew J. McBain, Catherine A. O’Neill

**Affiliations:** 1 Division of Musculoskeletal and Dermatological Sciences, School of Biological Sciences, Faculty of Biology Medicine and Health, The University of Manchester, Manchester, United Kingdom; 2 Bioimaging Core Facility, School of Biological Sciences, Faculty of Biology Medicine and Health, The University of Manchester, Manchester, United Kingdom; 3 Division of Pharmacy and Optometry, School of Health Sciences, The University of Manchester, Manchester, United Kingdom

**Keywords:** biofilm, keratinocyte, Lacticaseibacillus rhamnosus GG, skin, Staphylococcus aureus

## Abstract

*Staphylococcus aureus* is a leading cause of skin and soft tissue infections and a major nosocomial pathogen, with biofilm formation contributing to significantly higher morbidity and mortality. In atopic dermatitis (AD), where skin barrier dysfunction and microbial dysbiosis are common features, *S. aureus* abundance and biofilm formation are associated with disease severity and flare recurrence. We previously demonstrated that a lysate of *Lacticaseibacillus rhamnosus* GG (LGG)- a common human commensal and widely used probiotic strain- can inhibit *S. aureus* attachment to human keratinocytes by mechanisms including displacement and competitive exclusion. In this study, we extended these findings utilizing organ-cultured human skin to evaluate the protective effects of LGG lysate against *S. aureus* challenge in a physiologically relevant model. LGG lysate preserved skin integrity by preventing *S. aureus* penetration, proliferation, eDNA release and biofilm maturation. These effects were dose-dependent and effective when the lysate was applied from 24 hours pre-*S. aureus* inoculation to up to 3 hours post inoculation. Together, these data provide mechanistic insight into microbiome- pathogen interactions at the skin surface which could be exploited for therapeutic potential, particularly in the prevention of nosocomial *S. aureus* skin infections and mitigating *S. aureus*-driven flares in AD.

## Introduction

*Staphylococcus aureus* is an opportunistic pathogen capable of causing a range of diseases from superficial folliculitis to severe, life-threatening infections such as sepsis and endocarditis (Tong et al., 2015; Bashabsheh et al., 2024). Hospital-acquired *S. aureus* infections markedly increase patient morbidity and mortality and are associated with prolonged hospital stays, particularly among individuals undergoing surgery or those with indwelling medical devices (Singh et al., 2026; Ben-Amram et al., 2024; Brandquist and Kielian, 2025). These infections are exacerbated by the organism’s ability to deploy robust resistance mechanisms, including the formation of biofilms that enhance persistence and reduce antimicrobial susceptibility. Decolonization of the nose and skin using antibiotics or antiseptics remains the primary strategy for reducing *S. aureus* load to prevent subsequent infections (Piewngam and Otto, 2024).

*S. aureus* is also the predominant driver of infection-induced atopic dermatitis (AD), where disease flares are accompanied by temporal shifts in cutaneous microbial diversity and a pronounced increase in *S. aureus* abundance, which correlates with disease severity (Kong et al., 2012; Saheb Kashaf et al., 2023). Notably, *S. aureus* strains isolated from patients with severe AD are more likely to produce biofilm than those associated with mild AD, and higher biofilm propensity is strongly associated with skin barrier dysfunction (Gonzalez et al., 2019). Current therapeutic approaches to AD aim to reduce *S. aureus* burden, most commonly through antibiotic use. However, recurrent infections are likely, and repeated antibiotic exposure accelerates the development of antimicrobial resistance (Elizalde-Jiménez et al., 2024; Geoghegan et al., 2018; Miller et al., 2015). Furthermore, antibiotics exert broad, nonspecific effects on microbial communities, often depleting beneficial commensals alongside pathogenic species, resulting in long-lasting microbial disruption and increased susceptibility to dysbiosis-associated complications (De Nies et al., 2023; Baldanzi et al., 2026).

Together, these challenges highlight the urgent need for new, targeted, microbiome-conserving interventions capable of selectively disrupting *S. aureus* pathogenesis while preserving microbial communities. Given that biofilm formation contributes to persistence, immune evasion, antimicrobial resistance, and skin barrier dysfunction, biofilm developmental pathways present an especially promising therapeutic target for *S. aureus* infections.

The use of Lactic Acid Bacteria (LAB) as a strategy to combat infections is gaining increasing attention. LAB have long been recognized for their ability to support and stabilize gut microbiota through multiple mechanisms, including the production of organic acids and a range of antimicrobial products (Arunachalam et al., 2000; Pedone et al., 2000; Saavedra et al., 1994; Gui et al., 2025). Interest has expanded beyond gastrointestinal applications toward cutaneous use of LAB and their derivatives for chronic wounds, burns and skin ageing (Argañaraz Aybar et al., 2022; Jones et al., 2012; Falholt Elvebakken et al., 2023; Peral et al., 2010, 2009). Although only explored in a small number of studies, LAB have demonstrated the potential to interfere with *S. aureus* biofilms (Melo et al., 2016; Ahn et al., 2018; Saidi et al., 2023). Yet to date, no studies have demonstrated the anti-biofilm effects of LAB in human skin.

We previously demonstrated that a lysate of the LAB, *Lacticaseibacillus rhamnosus* GG (LGG) protects keratinocytes from the toxic effects of *S. aureus in vitro.* The protective mechanisms included the inhibition of *S. aureus* growth as well as competitive exclusion and displacement of the pathogen from keratinocytes (Mohammedsaeed et al., 2014; El-Chami et al., 2022). Building on these findings, we investigate the protective capacity of LGG lysate in a more physiologically relevant model – *ex vivo* human skin explants. As the mechanisms identified *in vitro* are central for early biofilm establishment, we further evaluate the ability of LGG lysate to interfere with *S. aureus* biofilm formation on skin explants.

## Materials and methods

### Bacteria and preparation of bacterial lysates

*Lacticaseibacillus rhamnosus* Goldin-Gorbach (LGG) obtained from the America Type Culture Collection (ATCC 53103, Manassas, USA) was grown anaerobically in Wilkins-Chalgren broth until the stationary phase. LGG lysates were prepared as described in Mohammedsaeed et al (Mohammedsaeed et al., 2014). The *S. aureus* isolate used was previously isolated from a chronic diabetic foot wound as described in Oates et al (Oates et al., 2012).

### *Ex vivo* skin organ culture

For most experiments, human skin was obtained from healthy adults undergoing liposculpture procedures. The study was approved by the North west Research Ethics Committee (REC ref. 14/NW/0185). All patients gave written informed consent. The skin was submerged in William’s E medium supplemented with 1% (v/v) L-glutamine, 0.02% (v/v) hydrocortisone and 0.1% (v/v) insulin (Thermo-Fischer, UK) for three days to wash out the disinfectant used during the surgery. Three, 4 mm biopsy punches per donor were then collected using a biopsy punch (Integra™ Miltex™, Fisher Scientific, Loughborough, UK) and placed in Thincert™ cell culture inserts with a pore size of 0.4 μm (Greiner Bio-One, Gloucestershire, UK). The insert was then placed in a 6-well plate such that the epidermis of the explant was exposed to the air and the dermis was submerged in medium. NativeSkin access (8 mm) biopsies (Genoskin, France) from healthy human donors were used for experiments visualizing biofilm formation on the skin surface using confocal microscopy. Biological replicates represent biopsies from individual donors.

### *S. aureus* inoculation and LGG lysate treatment of human skin explants

*S. aureus* was grown to exponential phase before inoculating the skin surface with 3x10^6^ colony forming units (CFU) in liquid suspension. A time-course of *S. aureus* biofilm formation on *ex-vivo* skin was visualized after incubation for 1, 3, 5, or 24 h. Alternatively *ex-vivo* skin explants were either: treated with 0.2 μg/mm^2^ of LGG lysate and simultaneously inoculated with 3x10^6^ CFU of *S. aureus*, treated with 0.2 μg/mm^2^ of LGG lysate for 1, 3, 5, or 24 h prior to inoculation with 3x10^6^ CFU of *S. aureus*, or inoculated with 3x10^6^ CFU of *S. aureus* for 1, 3, 5 or 24 h prior to treatment with 0.2 μg/mm^2^ of LGG lysate. The biopsies were incubated for 24 h at 37 °C in a humidified atmosphere of 5% CO_2_. Biopsies were then either embedded in Optical Cutting Temperature medium (OCT -Thermo-Fisher UK) and snap frozen for Fluorescence *in situ* hybridization (FISH) experiments or mounted on the confocal microscope (SP8 Leica upright confocal microscope) stage for live imaging of bacteria.

### *S. aureus* enumeration on human skin explants

The surface of the infected human skin explants was swabbed using a foam head swab (VWR International Ltd., Leicestershire, UK) 24 h post inoculation. The swab was immediately immersed in 100 μL of sterile PBS (pH 7.4) (Gibco, Thermo Fisher Scientific). The *S. aureus* viable count was performed by serial dilution plating and colony counting. After culture, the biopsies were snap-frozen in liquid nitrogen and stored at -80 °C.

### Fluorescence *in situ* hybridization for the detection of *S. aureus* in human skin explants

OCT embedded biopsies were sectioned using a cryostat and 5 μm frozen sections mounted on microscope slides. These were air-dried for 10 min, before being fixed at 80°C for 3 min and rinsed with 100% methanol. The slides were then fixed with 4% (w/v) PFA at 4°C for 10 min and rinsed in 100% methanol. Slides were left to air dry for 1 min and were then incubated with the lysis solution (20 mM Tris-HCl pH 7.0, 15 mg/mL lysozyme, 100 μg/mL lysostaphin) at 40°C for 8 min, to permeabilize the cells. Following this, the slides were rinsed in 100% methanol and air-dried for 1 min. Prewarmed hybridization solution (20 mM Tris-HCl pH 8.0, 0.9 mol/L NaCl, 20% formamide (v/v), 0.02% (v/v) SDS) was mixed with 0.5 μg/mL DAPI and 2 μM of the DNA probe specific for *S. aureus* (Biomer, Germany, Saur 16S69: 5’- GAA GCA AGC TTC TCG TCC G -3’) (38) and carefully applied to the tissue sections. The probe was 5′-end labeled with Atto-594 (Biomers, Germany). The slides were incubated in a dark and humid chamber at 47°C for 8 min, which was sufficient for the hybridization of the probes. The slides were then incubated in a pre-warmed wash buffer (0.318 mol/L NaCl, 20 mM Tris-HCl pH 8.0, 0.01% SDS, 10 mM EDTA) at 47°C for 1 min, then rinsed with PBS for 1 min and mounted with Fluoromount (Sigma-Aldrich, UK).

### Live/Dead staining of bacteria on human skin explants

3x10^6^ CFU *S. aureus* was applied for to the surface of human skin and incubated for 24 h. Following incubation, the bacteria were stained with the Live/Dead BacLight kit (Thermo Fisher Scientific, UK) for 15 min in the dark according to manufacturer instructions. Images were obtained on an SP8 Leica upright confocal laser scanning microscope using a 63×/0.9 water immersion objective. Images were analysed using GraphPad Prism v7.0 software (GraphPad Software Inc., CA, USA).

### Visualization of *S. aureus* biofilms on human skin explants

Biofilms were stained with the eDNA- specific cell-impermeant nucleic acid stain TOTO-1 (2 μM for 5 min, at 37oC) (Thermo Fisher Scientific, MA, USA) and counterstained with the cell-permeant nucleic acid stain SYTO-60 (10 μM for 15 min, at 37oC) (Thermo Fisher Scientific, MA, USA). Samples were visualized using excitation lasers of 513 nm and 650 nm for TOTO-1 and SYTO-60 respectively. An emission band of 520–550 nm was acquired for TOTO-1 and an emission band of 665–700 nm was acquired for SYTO-60. Biofilms were observed on an SP8 Leica upright confocal laser scanning microscope using a 63×/0.9 water immersion objective, performing z-stacks on selected regions.

Image analysis was performed using Imaris software (version 10.2; Bitplane, Zurich, Switzerland). TOTO-1 signal was quantified using the “Spots” function to detect discrete eDNA-associated puncta, while SYTO-60 signal was segmented using the “Surfaces” function to define total bacterial cells. The ratio of TOTO-1 spot count to SYTO-60 surface volume was calculated to assess relative eDNA abundance within the biomass.

### Statistical analysis

All experiments were carried out with three or more biological replicates, which are plotted as individual points on graphs. Graphical data are presented as mean of biological replicates ± SEM using GraphPad Prism 10 software. Results were considered statistically significant if P-values were less than 0.05. All statistical tests were carried out using GraphPad Prism 10 software. Parametric single-factor data with two groups was analysed using the unpaired *t* test. Single-factor data with three or more groups was tested using one-way ANOVA followed by multiple comparisons to a control group using Dunnett’s *post hoc* test.

## Results

### LGG lysate prevents *S. aureus-*induced tissue disruption of human skin

LGG lysate was previously demonstrated to protect the viability of keratinocyte monolayers from the toxic effects of *S. aureus*. Thus, we investigated whether the lysate could protect *ex-vivo* human skin from the effects of the pathogen.

**Figure 1:**
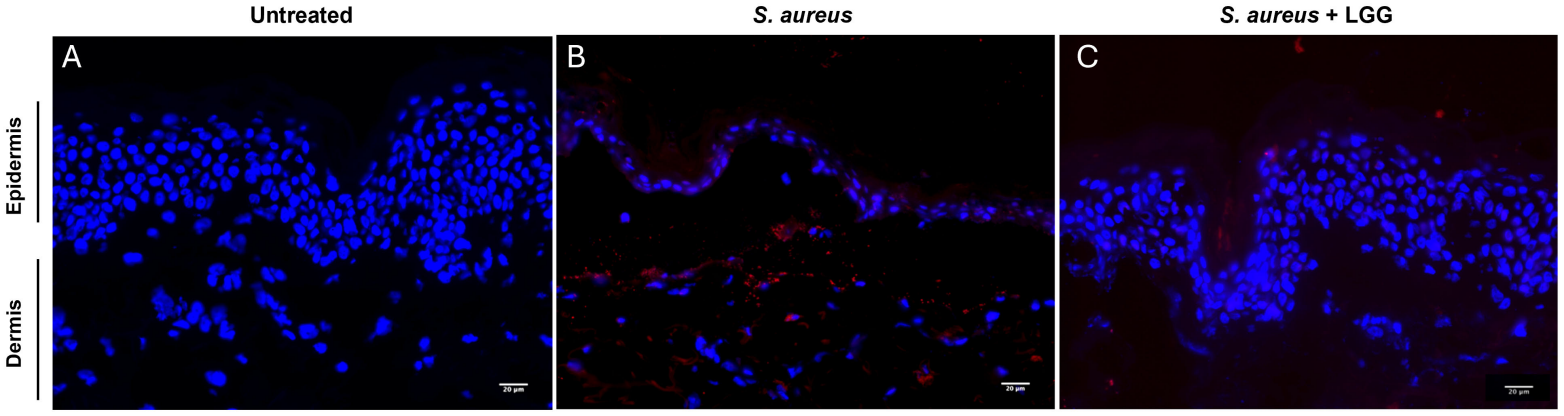
LGG lysate protects *ex vivo* human skin from the effects of *S. aureus. Ex vivo* human skin in organ culture was either **(A)** untreated, **(B)** treated with *S. aureus* for 24h or **(C)** treated with *S. aureus* in the presence of 0.2µg/mm^2^ LGG lysate for 24 h. Fluorescence *In Situ* Hybridization (FISH) was used to visualize *S. aureus* (red) penetration. The nuclei of skin cells were counterstained with DAPI (blue). Scale bar= 20µm.

Skin explants were simultaneously treated with 0.2 µg/mm^2^ LGG lysate and inoculated with 3x10^6^ CFU *S. aureus*. Following 24 h incubation, FISH targeting the *S. aureus* 16S rRNA gene was performed to visualize bacterial localization and assess tissue integrity. Fluorescence imaging of inoculated skin revealed that *S. aureus* caused marked separation of the epidermis from the dermis at 24 h post-inoculation (**Figure 1B**), and bacteria were observed translocating through the epidermis into the dermal compartment (**Figure 1B**). In contrast, application of 0.2 µg/mm^2^ LGG lysate preserved epidermal structure and prevented *S. aureus* penetration (**Figure 1C**). The lysate-treated skin appeared comparable to the untreated control skin, with no detectable structural disruption (**Figure 1A**).

### LGG lysate reduces *S. aureus* viable count on human skin explants in a dose- and time-dependent manner

**Figure 2:**
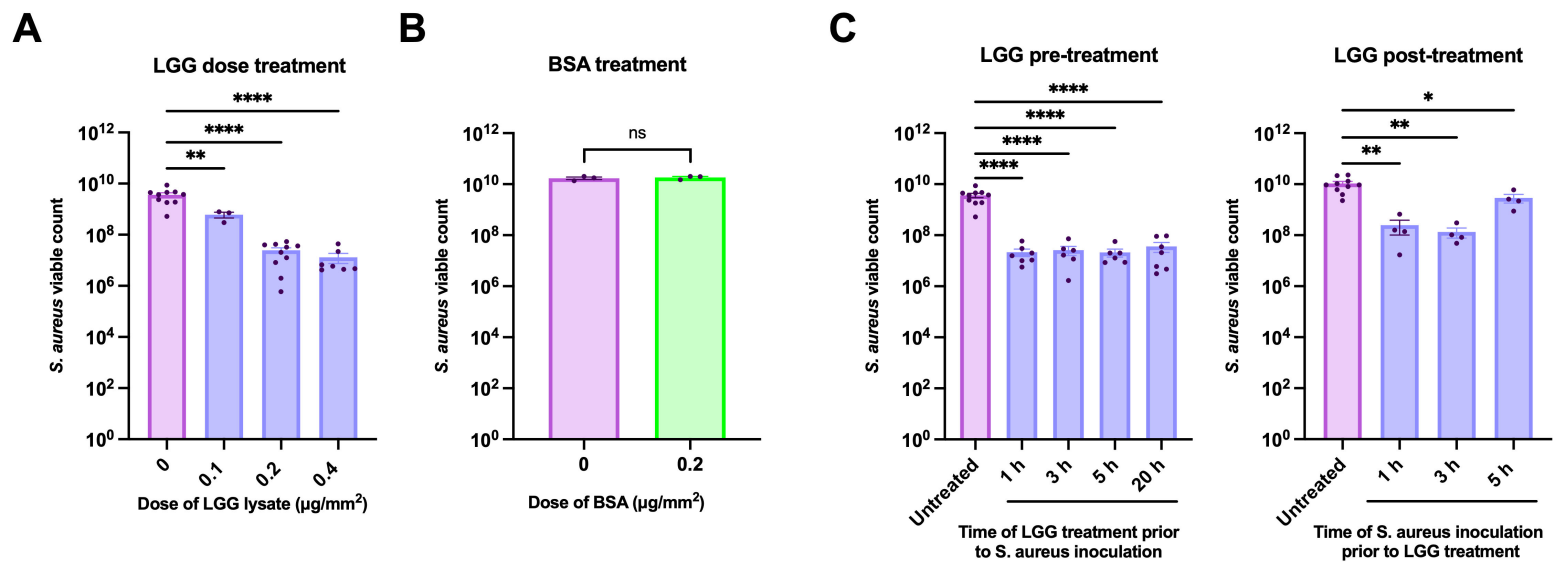
Dose and time dependent effects of LGG lysate on *S. aureus* viability on human skin explants. Application of 3x10^6^ CFU of *S. aureus* in the presence of 0.1 µg, 0.2 µg and 0.4 µg LGG lysate per mm^2^ resulted in a significant decrease in *S. aureus* viable count on the skin surface after 24 h of incubation, n≥3. **(B)** *Ex vivo* human skin explants treated with 0.2µg/mm^2^ of BSA exhibited no change in bacterial viable count compared to the control, n=3. **(C)** Treatment with LGG lysate at 1, 3, 5 and 20 hours prior to inoculation with *S. aureus* resulted in a significant reduction in the pathogen viable count. Skin explants treated with 0.2µg/mm^2^ of LGG lysate at 1 and 3 (but not 5) hours post-inoculation with *S. aureus* showed a significant reduction in bacterial viable count, n≥4. Data are represented as mean ± SEM, P values determined by **(A, C)** one-way ANOVA or **(B)** unpaired t-test, *p ≤ 0.05; **p ≤ 0.01; ****p ≤ 0.0001.

To assess whether the protective effects of the LGG lysate were associated with reduced *S. aureus* burden on the skin surface, we performed viable count assays. Human skin explants were treated with 0.1, 0.2 or 0.4 µg/mm^2^ LGG lysate and simultaneously inoculated with 3x10^6^ CFU *S. aureus*. After 24 h, all lysate doses produced a significant reduction in *S. aureus* viable counts compared with the untreated control. However, increasing the dose above 0.2 µg/mm^2^ did not enhance the magnitude of this effect (**Figure 2A**). In contrast, treatment with 0.2 µg/mm^2^ bovine serum albumin (BSA) had no effect on *S. aureus* viable counts (**Figure 2B**).

We next investigated whether the timing of lysate application influenced *S. aureus* viability. Pre-treatment of skin with 0.2 µg/mm^2^ LGG lysate for 1, 3, 5 or 24 h prior to inoculation with *S. aureus* resulted in a significant reduction in bacterial viable counts compared with untreated controls (**Figure 2C**). Similarly, application of LGG lysate at 1 or 3 h post-inoculation also resulted in a significant decrease in *S. aureus* recovery (**Figure 2C**). However, addition of LGG lysate at 5 h post-inoculation had no detectable effect on total *S. aureus* viable counts (**Figure 2C**).

### LGG lysate inhibits *S. aureus* growth on human skin explants

**Figure 3:**
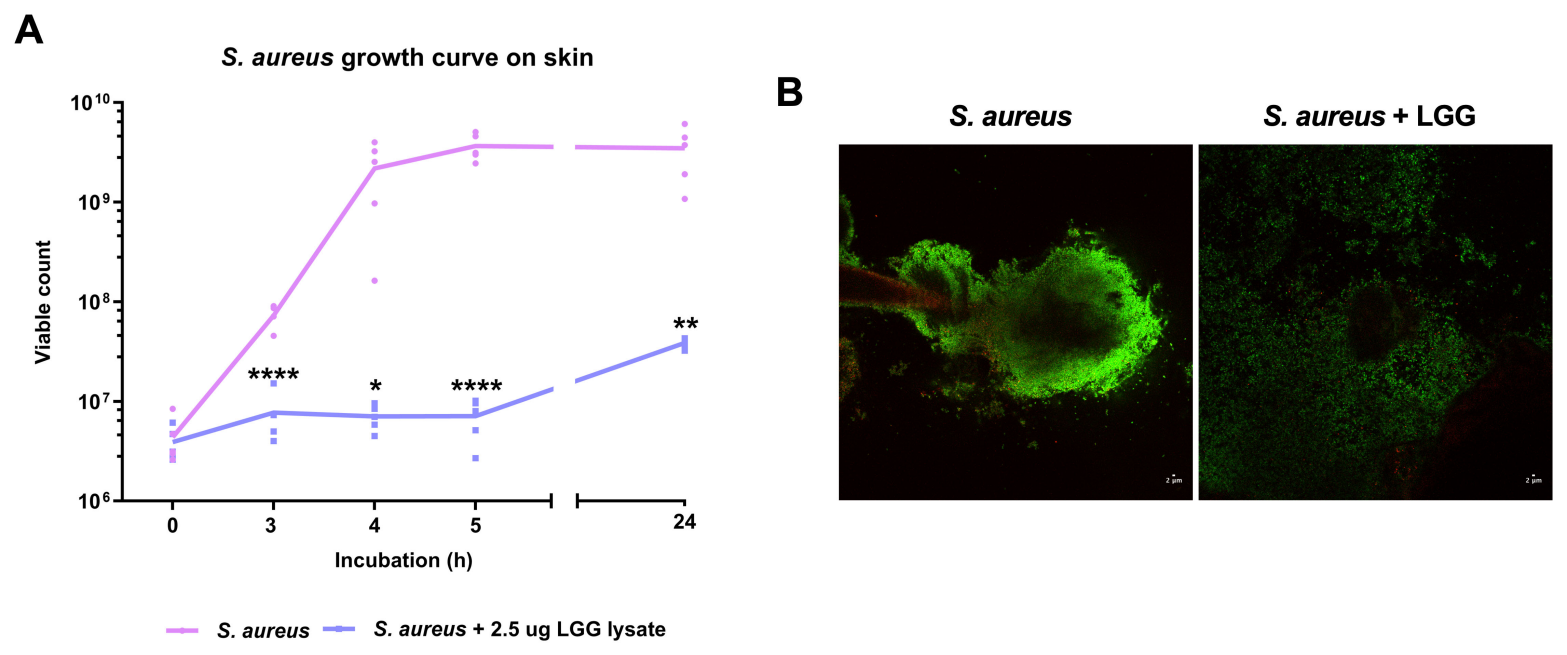
Effects of LGG lysate on *S. aureus* growth and morphology on human skin explants. **(A)** *S. aureus* growth curve on human skin in the presence and absence of 0.2 µg/mm^2^ LGG lysate, n=5. **(B)** Live/Dead stain of *S. aureus* on *ex-vivo* skin explants in the absence and presence of LGG lysate. Scale bars = 2um. Data are represented as mean ± SEM, P values determined by unpaired t-test *p ≤ 0.05; **p ≤ 0.001; ****p ≤ 0.0001.

To determine whether the reduced *S. aureus* viable counts observed in the presence of LGG lysate were due to inhibition of bacterial growth, we performed growth curves of *S. aureus* on the surface of the human skin explants. In the absence of LGG lysate, *S. aureus* proliferated on the *ex vivo* skin and reached stationary phase approximately 5 h post-inoculation (**Figure 3A**). In contrast, treatment of 3x10^6^ CFU of *S. aureus* with 0.2 μg/mm^2^ of LGG lysate significantly inhibited *S. aureus* growth during the initial 5 h of inoculation and resulted in a significant reduction in viable counts after 24 h compared with untreated controls (**Figure 3A**).

Consistent with these quantitative findings, golden *S. aureus* colonies were easily visible on the surface of untreated explants 24 h post-inoculation, while they were far less apparent on LGG lysate-treated explants (**Supplementary Figure 1**). Live/Dead staining revealed no increase in the number of dead cells in the presence of the lysate; however, notable morphological differences were observed. In the absence of LGG lysate, *S. aureus* formed dense aggregates on the skin surface, while lysate treatment resulted in a more dispersed, distribution of cells (**Figure 3B**).

### LGG lysate prevents biofilm formation by *S. aureus* on human skin explants

**Figure 4:**
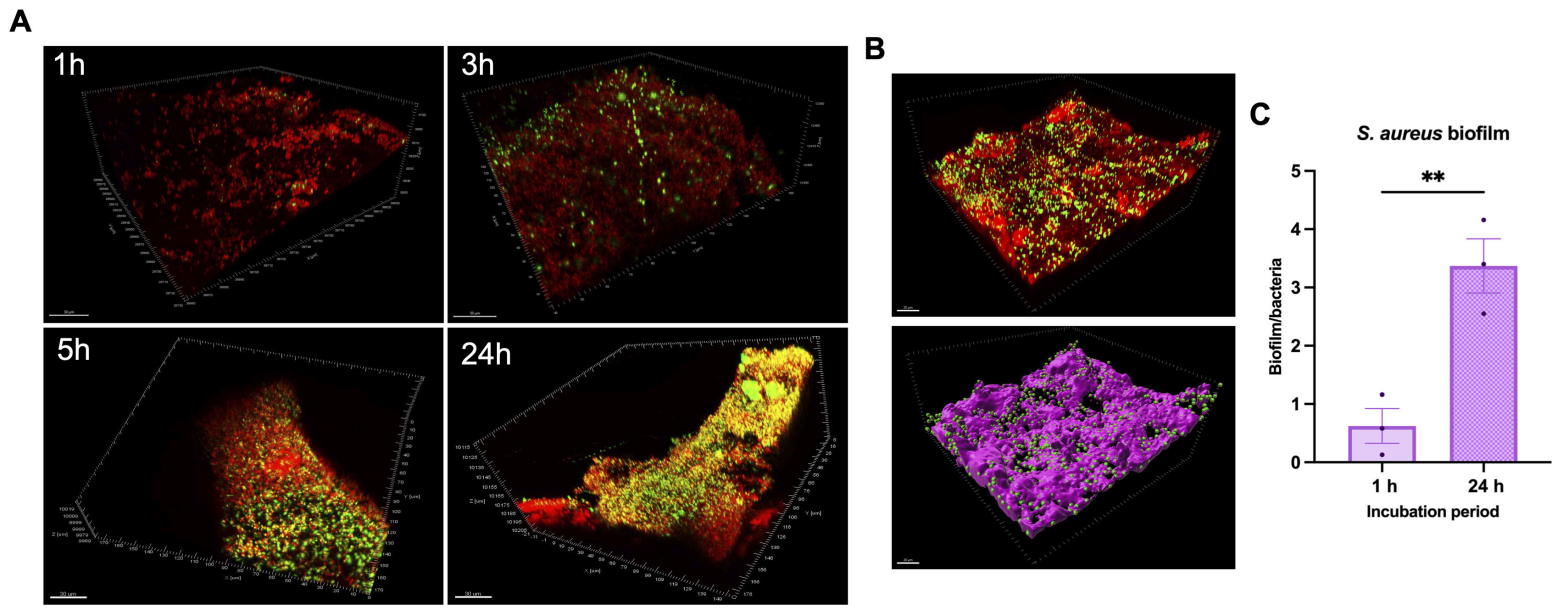
Time course of *S. aureus* biofilm formation on *ex vivo* human skin. **(A)** Confocal microscopy visualization of extracellular DNA (eDNA) staining with TOTO-1 (green) and *S. aureus* nuclear counterstaining with SYTO 60 (red) in *S. aureus* biofilms on human skin at 1, 3, 5 and 24 h post inoculation. **(B)** eDNA and bacteria were segmented separately as dots and surface respectively using IMARIS image analysis software to calculate the amount of biofilm relative to the bacterial load. **(C)** The amount of biofilm deposition significantly increased over the 24 h growth period. Data are represented as mean ± SEM, n=3, P values determined by unpaired t-test, **p ≤ 0.01.

The loss of *S. aureus* aggregation observed by Live/Dead staining prompted us to investigate whether LGG lysate affected biofilm formation. To assess this, we used immunofluorescence and confocal microscopy to visualize the presence of extracellular DNA (eDNA), a well-established marker of biofilm development (Alhede et al., 2020; Campoccia et al., 2021; Whitchurch et al., 2002). Fluorescent staining with TOTO-1 (green) to label eDNA and SYTO-60 (red) as a bacterial counterstain revealed a progressive accumulation of eDNA on the surface of *ex vivo* human skin over the 24 h incubation period, culminating in the formation of multi-layered bacterial aggregates (**Figure 4A**). To quantify the increase in biofilm deposition, IMARIS software was used to segment both fluorescent channels and calculate the amount of biofilm produced relative to the bacteria load (**Figure 4B**). This analysis showed a significant increase biofilm deposition after 24 h incubation (**Figure 4C**).

**Figure 5:**
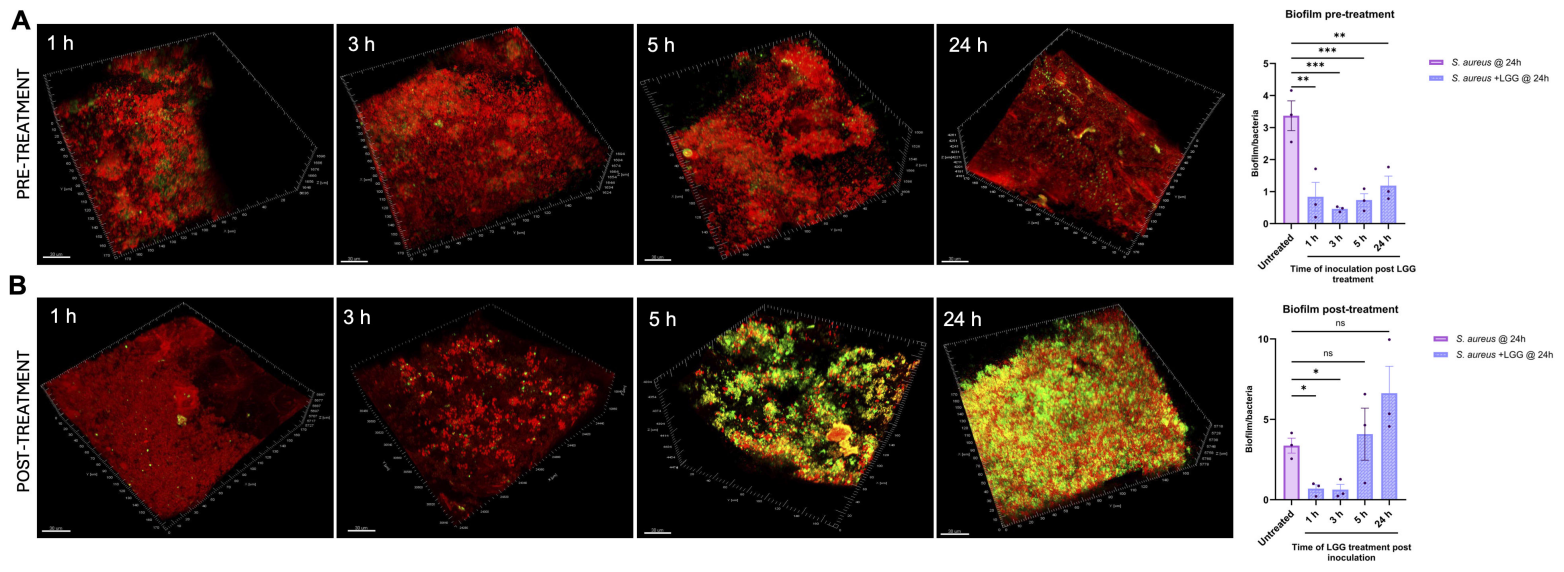
Pre- and post-treatment of *ex vivo* human skin with LGG lysate. Confocal microscopy visualization of extracellular DNA (eDNA) staining with TOTO-1 (green) and *S. aureus* nuclear counterstaining with SYTO 60 (red) in *S. aureus* biofilms on human skin 24 h post *S. aureus* inoculation **(A)** following pre-treatment with 0.2µg/mm^2^ LGG lysate for 1, 3, 5 and 24 h before inoculation, **(B)** or post-treatment after *S. aureus* inoculation. Data are expressed as biofilm relative to the bacterial load. Data are represented as mean ± SEM, n=3, P values determined by one-way ANOVA, *p ≤ 0.05; **p ≤ 0.01; ***p ≤ 0.001.

Pre-treatment of the *ex vivo* skin with 0.2 µg/mm^2^ LGG lysate for 1, 3, 5 or 24 h resulted in a significant reduction in eDNA accumulation (**Figure 5A**). Under these conditions, *S. aureus* formed negligible biofilm, presenting primarily as a uniform layer of cells with little to no aggregation. In contrast, post-treatment of established *S. aureus* biofilms with 0.2 µg/mm^2^ of LGG lysate had variable effects (**Figure 5B**). Application of LGG lysate 1h and 3h post-infection significantly reduced eDNA levels, whereas treatment at 5 h and 24 h post-inoculation did not substantially inhibit biofilm development, as evidenced by the presence of extensive eDNA deposition (**Figure 5B**).

## Discussion

The use of LAB for skin health is an emerging area of research, with increasing focus on the potential use of topical LAB or their extracts to improve skin function and treat dermatological conditions (Deng et al., 2024; Łętocha et al., 2025; Lebeer et al., 2022). Patients with AD typically exhibit elevated *S. aureus* abundance on their skin compared with healthy individuals, particularly during disease flares, and multiple studies have reported a correlation between *S. aureus* burden and AD severity (Lipnharski et al., 2013; Gonzalez et al., 2019; Tauber et al., 2016; Byrd et al., 2017; Kong et al., 2012). These observations support a growing consensus that *S. aureus* contributes to AD pathogenesis and that strategies aimed at reducing cutaneous *S. aureus* colonization may help alleviate AD symptoms and improve disease management. Beyond AD, *S. aureus* is a leading cause of hospital acquired infections and there is an urgent need for effective preventative measures. Ideally, strategies aimed at limiting pathogenic activity should avoid bactericidal mechanisms, as selective pressure can promote the emergence of resistance or escape mutants (Oz et al., 2014). Instead, approaches that interfere with virulence factors offer a promising alternative. Clinical isolates of *S. aureus* taken from both AD patients and hospital acquired infections are often biofilm-positive, and biofilm formation is known to contribute to antimicrobial tolerance and immune evasion, resulting in persistent, high-morbidity infections (Oates et al., 2014, Ben-Amram et al., 2024; Gonzalez et al., 2019; Elizalde-Jiménez et al., 2024). Consequently, interventions that inhibit biofilm development without exerting selective pressure through bacterial killing may represent an effective prophylactic strategy for individuals with AD as well as patients within healthcare settings.

Using *ex vivo* human skin maintained in organ culture, we demonstrate that topical application of an LGG lysate protects the skin from the detrimental effects of *S. aureus*. In the presence of LGG lysate, normal epidermal architecture was preserved, and *S. aureus* penetration into deeper layers of the skin was prevented (**Figure 1**). The lysate did not exert bactericidal activity, as viable counts remained comparable to the initial inoculum (**Figure 2**), and Live/Dead staining confirmed that most cells remained viable (**Figure 3B**). However, the lysate significantly suppressed *S. aureus* proliferation (**Figure 3A**) and impaired biofilm development, evidenced by a significant reduction in eDNA accumulation (**Figure 5**). These effects were attributable to specific bioactive components within the lysate rather than nonspecific protein content, as bovine serum albumin had no effect on *S. aureus* viability (**Figure 2B**).

The effects of the lysate were time-dependent; while it was protective when applied prior to challenge with the pathogen, addition of the lysate later than 3 h post-challenge failed to inhibit biofilm formation (**Figure 5**). In our previous work, we showed that LGG lysate prevented *S. aureus* adhesion to keratinocytes and was capable of displacing the pathogen when applied either before or after bacterial challenge. However, in the more complex environment of human skin, this capacity is more limited. Unless the lysate is applied within the first 3 h following *S. aureus* inoculation, eDNA release and biofilm maturation proceed unabated.

eDNA is a major structural component of the biofilm matrix in numerous bacterial species and plays a fundamental role in biofilm establishment, particularly during the early stages of development (Whitchurch et al., 2002). In *S. aureus* biofilms, the incorporation of eDNA into the biofilm matrix has been shown to depend on the presence of secreted matrix proteins, whereas the secretion of matrix proteins occurs independent of eDNA. Current hypotheses therefore propose that matrix proteins first associate with the bacterial cell surface, after which eDNA is produced and incorporated into the developing biofilm (Dengler et al., 2015). The *S. aureus* biofilm matrix consists of extracellular proteins, polysaccharides, and DNA, although the specific mechanistic contributions of each component remain incompletely understood. Notably, some studies suggest that *S. aureus* utilizes “moonlighting” proteins as part of its extracellular matrix (Foulston et al., 2014).

Moonlighting proteins are usually proteins of central metabolism that relocate to the cell surface to function as adhesins (Kainulainen and Korhonen, 2014). LGG lysate contains similar moonlighting proteins, and we have previously shown that they can inhibit *S. aureus* adhesion to keratinocytes in culture (El-Chami et al., 2022). Thus, our data suggest that the mechanism utilized by LGG lysate may involve competitive inhibition of the interactions of *S. aureus*-derived moonlighting proteins with the skin surface, which then prevents eDNA associating with the matrix and hence, reduction in biofilm formation. Failure of these proteins to bind prevents the subsequent incorporation of eDNA into the extracellular matrix, thereby impairing biofilm maturation. This proposal is further supported by our observations that LGG lysate can only inhibit biofilm formation but cannot disrupt an already established biofilm seen after 5 h incubation (**Figures 4A**, **5**). The loss of inhibitory activity at 5 h likely reflects occupation of the relevant binding sites by *S. aureus-*derived proteins, making them inaccessible to proteins from the lysate and permitting continued biofilm development (**Figure 5B**).

The potential of probiotics to manage biofilm-associated infections has previously been reviewed (Barzegari et al., 2020). Postbiotics, such as bacterial lysates, offer several advantages over live probiotics as they eliminate the risks associated with administering viable bacteria, have enhanced stability, and are more compatible with topical formulations. The *S. aureus* strain used in this study was a clinical isolate obtained from a biofilm-positive chronic diabetic foot wound. The bacteriostatic and anti-biofilm activity of the LGG lysate against this pathogenic isolate emphasizes its potential as a protective or adjunctive therapeutic strategy for the management of chronic, biofilm-associated wounds.

Pre-treatment of *ex vivo* skin for 24 h prior to *S. aureus* challenge further demonstrates both the stability and the prophylactic potential of the LGG lysate, indicating its suitability as a preventative measure. In a previous study, we also showed that LGG lysate enhances the expression of key epidermal barrier proteins in keratinocytes (Sultana et al., 2013). Since AD is characterized by both increased *S. aureus* colonization and impaired barrier integrity, interventions capable of simultaneously limiting *S. aureus* activity and strengthening the skin the barrier- such as LGG lysate- may be particularly advantageous for preventing or attenuating the severity of AD flares.

A limitation of this study is the use of a single *S. aureus* isolate. Although derived from a chronic diabetic foot ulcer, representing a clinically relevant, biofilm-associated infection niche in which biofilm formation underpins persistence and treatment failure, this approach may not fully capture the strain-to-strain variability in biofilm formation across *S. aureus* lineages. Future work should extend these findings across a diverse panel of clinically isolated strains to determine the broader applicability of the observed effects.

Collectively, these results demonstrate that LGG lysate can attenuate *S. aureus* pathogenicity by suppressing bacterial proliferation and preventing biofilm establishment. This dual activity highlights its potential as a prophylactic or adjunctive strategy for preventing chronic, biofilm-associated infections and AD flares.

## Data Availability

The original contributions presented in the study are included in the article/**Supplementary Material**. Further inquiries can be directed to the corresponding author.

## Supplementary material

Supplementary Figure 1 LGG lysate visibly reduces *S. aureus* growth on the skin surface. Both biopsies were inoculated with 3x10^6^ CFU *S. aureus.* One biopsy was simultaneously treated with 0.2µg/mm^2^ LGG lysate, while the other was left untreated. Images were captured after 24 h incubation at 37 °C in a humidified atmosphere of 5% CO_2_. The LGG lysate-treated biopsy showed visibly reduced *S. aureus* growth compared with the untreated biopsy, which displayed characteristic yellow colonies.
